# Validation of nasospheroids to assay CFTR functionality and modulator responses in cystic fibrosis

**DOI:** 10.1038/s41598-021-94798-x

**Published:** 2021-07-30

**Authors:** Maite Calucho, Silvia Gartner, Paula Barranco, Paula Fernández-Álvarez, Raquel García Pérez, Eduardo F. Tizzano

**Affiliations:** 1 Medicine Genetics Group, Vall D’Hebron Research Institute, 08035 Barcelona, Spain; 2 Department of Clinical and Molecular Genetics, Hospital Universitari Vall d’Hebron , 08035 Barcelona, Spain; 3 Cystic Fibrosis Unit, Hospital Universitari Vall d’Hebron, 08035 Barcelona, Spain; 4 Autonomous University of Barcelona, 08193 Bellaterra, Barcelona, Spain

**Keywords:** Respiratory system models, Predictive markers, Cystic fibrosis

## Abstract

The availability of a simple, robust and non-invasive in vitro airway model would be useful to study the functionality of the cystic fibrosis transmembrane regulator (CFTR) protein and to personalize modulator therapy for cystic fibrosis (CF) patients. Our aim was to validate a CFTR functional study using nasospheroids, a patient-derived nasal cell 3D-culture. We performed live-cell experiments in nasospheroids obtained from wild-type individuals and CF patients with different genotypes and phenotypes. We extended the existing method and expanded the analysis to upgrade measurements of CFTR activity using forskolin-induced shrinking. We also tested modulator drugs in CF samples. Immobilizing suspended-nasospheroids provided a high number of samples for live-cell imaging. The diversity observed in basal sizes of nasospheroids did not affect the functional analysis of CFTR. Statistical analysis with our method was simple, making this protocol easy to reproduce. Moreover, we implemented the measurement of inner fluid reservoir areas to further differentiate CFTR functionality. In summary, this rapid methodology is helpful to analyse response to modulators in CF samples to allow individualized treatment for CF patients.

## Introduction

Cystic fibrosis (CF) is the most common lethal monogenic disease with an incidence of 1/3000–10,000 newborns. It is caused by bi-allelic pathogenic variants in the cystic fibrosis transmembrane regulator (*CFTR*) gene and codifies for an epithelial Cl^−^ channel ^1^. More than 2000 pathogenic variants have been reported in the *CFTR* gene to date but the F508del pathogenic variant is present in about 65% of mutant alleles worldwide ^2^. *CFTR* pathogenic variants are classified into seven types (I-VII) according to their effect on the CFTR protein (Supplementary Table S1) ^3^. In view of the wide diversity of genotypes, clinical severity is variable. However, the most common and life-threatening manifestation is lung disease leading to respiratory failure.

The recent development of CFTR modulator drugs – correctors and potentiators – has been successful in partially restoring the CFTR protein defect. Corrector molecules (VX-809 lumacaftor, VX-661 tezacaftor, and VX-445 elexacaftor) partially restore the CFTR processing defects and the protein can reach the cell surface ^4,5^. The VX-770 potentiator (ivacaftor) enhances the CFTR channel opening (Supplementary Table S1) ^6^. Modulator drugs are now approved for several *CFTR* genotypes ^7–10^. Monotherapy with potentiator VX-770 (KALYDECO) is addressed to class III and IV genotypes ^6^. The combination lumacaftor with ivacaftor (ORKAMBI) has been used satisfactorily to treat patients who are homozygous for F508del, a class II pathogenic variant ^11^. Tezacaftor with ivacaftor (SYMDEKO/SYMKEVI) is indicated for patients who are F508del homozygous or F508del heterozygous with a residual function variant ^12,13^. More recently, a triple combination with two different correctors (tezacaftor and elexacaftor) and potentiator ivacaftor (TRIKAFTA/KAFTRIO), has been approved to treat F508del homozygous patients ^10,14^, or those who carry a single copy of the F508del genetic variant with a minimal function variant ^15,16^.

The advent of modulator therapy has undoubtedly advanced healthcare for CF patients. However, CFTR-drug restoration is limited in some patients ^17,18^ and new pharmacological approaches are currently under investigation to treat patients for whom no specific treatment is yet available, such as those with class I, VII or rare pathogenic variants. It is therefore crucial to study CFTR function individually in order to predict responses to currently approved or experimental drugs.

In this context, CFTR functional assays using in vitro patient-derived cells have emerged in recent years. The most remarkable approach is the forskolin-induced swelling (FIS) assay in intestinal organoids ^17^. This in vitro assay stratifies patients according to disease severity and predicts CFTR restoration after treatment ^19–22^. However, it requires invasive biopsy, time-consuming cell cultures, and highly skilled personnel. Additionally, this model is derived from intestinal epithelium which may not be specific to study airway CFTR function. In an attempt to solve this inconvenience, a variety of respiratory patient-derived cell models has been investigated ^23–29^. Along these lines, Guimbellot et al. ^30^ developed a simple model of nasospheroids, a 3D nasal cell culture obtained by means of a minimally invasive procedure. This nasal cell culture approach enables measurements of CFTR-fluid transport by live-cell assay, and is therefore a potential tool to test the efficacy of modulator drugs in CF patients. However, a standardized protocol is not yet well established and the approach has two main limitations: the number of nasospheroids available for live-cell assay is low and the statistical analysis is complex.

Our aim was to validate the use of nasospheroids and enhance their reproducibility for implementation in order to analyze CFTR function and to test CFTR modulator drugs. Moreover, we aimed to overcome some limitations of the original protocol by implementing modifications in both the technique and the posterior analysis.

## Methods

### Participants

The study was conducted at the Cystic Fibrosis Unit and facilities at the Vall d’Hebron Research Institute at Hospital Vall d’Hebron (Barcelona, Spain). The protocol was approved by the local ethics committee (Comité Ético de Investigación con Medicamentos y Comisión de Proyectos de Investigación del Hospital Universitari Vall d’Hebron) (PR(AMI)291/2017) and all methods were performed in accordance with relevant guidelines and regulations. Informed consent was signed by all participants, and their legal representative if under-age. Seven healthy wild-type (WT) volunteers over 18 years of age and 7 CF patients aged 6–19 years were recruited for this study. CF patients were diagnosed by positive neonatal screening, abnormal sweat chloride test and the presence of two pathogenic variants in the *CFTR* gene in all cases. CF patients were stable and recruited in a routine follow–up, and they presented the following genotypes:  F508del /2183AA>G (n = 2), G542X/R117H-7 T (n = 1),  F508del/3849+10kbC>T (n = 1), L227R/L227R (n = 1), F508del/F508del (n = 1) and F508del/1812-1G>A (n = 1). Patients sharing genotype  F508del/2183AA>G were unrelated. Pathogenic variants are indicated with their legacy names. Table 1 provides further information on patients’ demographic and clinical characteristics.

**Table 1:** Patients’ clinical information and in vitro response to modulators.

Subject (Gender)	Genotype	Functional class	Age at sampling procedure	Pancreatic function	FEV_1_%	Bronchiectasis	Chronic colonization	In vitro response to modulators
**CF1** (**M**)	F508del/2183AA>G	II/I	15	Insufficiency	45	Yes	SA-PA	** + + **
**CF2** (**F**)	F508del/2183AA>G	II/I	17	Insufficiency	85	Yes (mild)	No	** + **
**CF3** (**M**)	F508del/3849 + 10KbC>T	II/V	6	Sufficiency	110	No	No	+ + *
**CF4** (**M**)	L227R/L227R	Undetermined	19	Insufficiency	80	Yes	MRSA	**-**
**CF5** (**F**)	G542X/R117H-7H	I/IV	12	Sufficiency	116	No	No	**-**
**CF6** (**M**)	F508del/F508del	II	18	Insufficiency	57	Yes	SA-PA	** + + **
**CF7** (**M**)	F508del/1812-1G>A	II/I (canonical splicing)	18	Insufficiency	86	Yes	SA	**-**

CF1 and CF2 subjects share the same genotype but are unrelated. Pathogenic variants are indicated with their legacy names.

Gender: M (male) F (female); Chronic colonization: SA (*Staphylococcus aureus*), MRSA (methicillin resistant *Staphylococcus aureus*), SA-PA (*Staphylococcus aureus* and *Pseudomonas aeruginosa*). Last column describes in vitro nasospheroid response to VX-770 + VX-809 and/or VX-770 + VX-661 assessing FR and AUC analysis in IFRA after live-cell imaging assay (t = 60 min). Responses were categorized according to the statistical differences found between FSK and VX treatment: +  + notable response = observed in both, FR and AUC values; + moderate response = significant differences in FR or AUC values; − no response = no significant differences. * Only responder to VX-770 + VX-661.

### Nasal epithelial cell sampling

Nasal epithelial cells were collected by skilled professionals in the Cystic Fibrosis Unit of Hospital Vall d’Hebron using curettes (ASI Rhino-Probe, Arlington Scientific, Springville, UT). Two nasal curettages were collected from each nostril and cells were placed in 5 ml complete culture medium.

### Growth and expansion of nasal epithelial cells

We modified the original protocol from Guimbellot et al. for expansion and maintenance of nasospheroids in three ways ^30^. First, the culture medium used was bronchial epithelial cell medium (BEpiCM ScienceCell; Carlsbad, CA, USA) supplemented for 3 days with 0.33 nM EC23, 1 µM A83-01, 5 µM Y-27632 (72304 STEMCELL Technologies, Vancouver, Canada), and 1 µM DMH-1. Second, nasospheroids were grown in standard 24-well plates. And third, nasospheroids were embedded in Matrigel (Growth Factor Reduced and phenol-free, Corning, NY) under two conditions. In the standard protocol or condition 1 (C1), nasospheroids were grown and maintained in suspension until 24 h before the confocal live-cell imaging experiments*.* Cells were then collected in a 1.5 ml tube. After centrifugation (400 g, 5 min, 4 °C), 5 µl-drops of pellet were plated in a heated (37 °C, 1 week) 24-well black plate (ibidi GmbH 82406, Gräfelfing, Germany). Pellet drops were resuspended 1:1 with Matrigel and incubated for 15 min (37 °C) to let the Matrigel solidify before adding complete medium. For condition 2 (C2), fresh nasal epithelial cells were centrifuged (400 g, 5 min, 4 °C) and the pellet was slowly resuspended in Matrigel (final concentration 1:1). Matrigel drops were placed in standard 24-well plates. Cells were maintained until live-cell experiments were performed. Twenty-four hours before these experiments, nasospheroids were collected, centrifuged and transferred to 24-well black plates. In vitro live-cell imaging was carried out between days 8 and 10 after cell collection.

### Confocal live-cell microscopy image

Nasospheroids were labelled with 8 µM Calcein Green AM 15 min before starting the confocal live-cell experiments. For CFTR activation, we administered 10 µM forskolin (FSK) (F3917 Sigma-Aldrich; to increase cAMP levels), 100 µM amiloride (to inhibit epithelial sodium channel) and 100 µM 3-Isobutyl-1-methylaxanthine (IBMX) (to maintain intracellular cAMP levels)^30^. Modulators (VX-770, VX-809 and VX-661; S1144, S1565, S7059 Selleckchem) were tested at 5 µM and combined with FSK, Amiloride and IBMX. Correctors VX-809 and VX-661 were incubated for 24 h before the live-cell analysis. The vehicle control used was dimethyl sulfoxide (DMSO) (final concentration < 1% in culture medium; without supplements). CFTR inhibitor (CFTR_inh_ – 172; Sigma-Aldrich 219670) was incubated for 3 h (10 µM).

### Equipment and settings

Microscopy fields were manually chosen at basal conditions (before administering any compound). Five z-stacks at 6 µm distance were taken sequentially. Images of the selected sections were taken every 10 min (up to 60 min) using a Zeiss LSM780 confocal microscope. This microscope has a motorized stage and a chamber controlling CO_2_ (5%), humidity (55%) and temperature (37 °C); the chamber was preheated for 1 h. A 10 × 0.4 NA objective was used. Images were captured in bright-field and fluorescent/calcein emission (488 nm excitation and 500–530 nm emission). Images were obtained at 512 × 512, 8-bit. The confocal pinhole was set to 33.4.

### Image analysis

Areas of nasosopheroids were analyzed by ImageJ/FIJI ^31^ using maximum intensity projection images. The contour of the nasospheroids was manually delineated using the *freehand* tool. IMAGEJ/FIJI software automatically calculates the selected area. For inner areas, we delineated the area of the lumen using the same tool. This delineation have been illustrated in previous reports ^17,32^.

### GraphPad statistics

Statistical analysis tests based on the raw data were assessed using Prism statistical software version 6 and 8 (GraphPad) ^33^. Significance was considered at *p* < 0.05.

Area under the curve (AUC) values were calculated from raw data and based on intestinal organoid studies in CF research ^17^. Automatic AUC analysis (section “XY analyses”) were performed by GraphPad Prism software, applying baseline Y = 0.

### Development of a specific mixed-level model for analysis of nasospheroids

The logarithmic model was specifically designed for our data to analyze the area of WT nasospheroid according to “Stimulus” (FSK and DMSO) and “Condition” of cell culture (C1 or C2) (Supplementary Table S2). We assessed a novel mixed model for repeated measurements which were logarithmically transformed ^34^ (Supplementary Table S3). Supplementary Table S2 shows the variables considered for the model. All variables were considered independent and each nasospheroid was considered an aleatory factor. The level of significance used in the statistical tests was *p* < 0.05. Several model structures were explored using the Akaike information criterion (AIC) adjusted index. The initial model with logarithmic transformed measures showed an AIC value of − 445,479. To describe quantitative variables we used the mean, standard deviation, maximum, and minimum values. To describe qualitative variables we analyzed the absolute and relative frequencies. Statistical analysis was carried out using SAS v9.4 and R software v3.1.2. The individual component was incorporated as a random factor.

## Results

### Formation and embedding of nasospheroids for in vivo imaging analysis

We first validated the formation of WT nasospheroids in cell cultures from epithelial nasal cells obtained by nasal curettage. A total of 5–7 × 10^4^ cells were seeded, without resuspension, and cell aggregates are formed a few hours later. Three days after culture in a bronchial serum free-medium, these clusters developed hundreds of completely formed nasospheroids with the apical surface facing outward and expressing CFTR protein (Supplementary Fig. S1). Nasospheroid size depended on the number of agglutinated epithelial cells forming the spherical structure. CFTR FSK/cAMP-stimulated transport was studied in nasospheroids by live-cell imaging to analyze shrinking after CFTR activation ^30^. Given that cells were growing in suspension, we embedded nasospheroids in Matrigel to warrant stability and unambiguous follow-up during the live-cell imaging analysis. The maximum number of nasospheroids analyzed per experiment and sample was 55 (mean per experiment ± SD = 21.310 ± 16.112).

**Figure 1:**
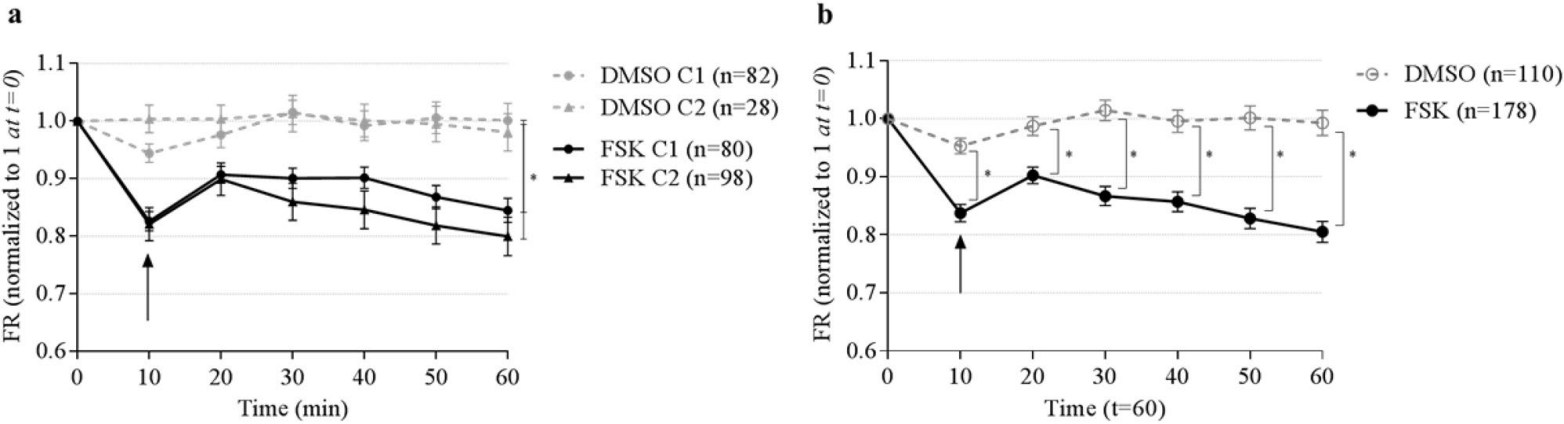
Similar responses of WT nasospheroids using two conditions of exposure to Matrigel and differences between incubation with FSK or DMSO*.* (**a**) Nasospheroids from seven WT patients were grown in two conditions: the standard protocol or *condition 1* (C1) (spheres formed in suspension and embedded in Matrigel the day before live-cell imaging) and *condition 2* (C2) (spheres in Matrigel for 7 days minimum) (see details in *Methods*). The starting size of each nasospheroid was set at 1. At time point 0, nasopheroids were in basal conditions. For CFTR activation (*FSK*), 10 µM forskolin plus IBMX and amiloride (for epithelial sodium channel “ENaC” inhibition) were incubated (t = 10; black arrow). For non-stimulated spheres, dimethyl sulfoxide (*DMSO*) was administered. Size reduction in respect to basal size was calculated along time points up to 60 min. No significant differences were found between conditions 1 and 2 (“FSK” incubation or non-stimulated “DMSO”) (*p = 0.1563; paired t-test). Dots and triangles represent the mean fractional reduction (FR) at each time point. Bars represent standard deviation error (SEM). (**b**) Results from spheres grown under conditions 1 and 2 were combined. Differences between stimulus DMSO and FSK were observed from t = 10 and increased over time (t-test by GraphPad Prism and by our newly developed logarithmic-transformed measures’ model) (t = 10 min *p* = 0.008, t = 35 min *p* = 0.0.0004, t = 70 min *p* = 0.0002). We also observed a reduction peak when DMSO or FSK were added at time 10 (black arrow). This change could be due to a reaction of nasospheroids to the addition of compounds. Bars represent standard deviation error (SEM). Graphic created with GraphPad Prism version 6^33^.

To rule out the influence of Matrigel in CFTR-dependent transport, we performed the experiments in two different conditions: C1*,* where nasospheroids were embedded in Matrigel the day before live-cell imaging, and C2, where spheres were grown embedded in Matrigel from the start (details in *Methods*) (Supplementary Fig. S2, Supplementary Video S1 and S2). Nasospheroids grown in both conditions presented different mean sizes (mean ± SD): 7911.351 ± 6774.84 µm^2^ (n = 162 for C1) and 5956.277 ± 5432.514 µm^2^ (n = 125 for C2) (***p = 0.0003 t-test). Changes in cross-sectional areas (CRA) of nasospheroids were calculated relative to t = 0 (fractional reduction, FR). After 60 min of FSK administration, we analyzed the FR of 80 nasospheroids in C1 and 98 in C2, and we found no significant differences between the two conditions (*p* = 0.1563; paired t-test) (Fig. 1a). Matrigel-embbeded nasospheroids (C1 and C2) presented similar FR mean values after FSK administration (t = 60) than suspended nasospheroids without Matrigel (Supplementary Fig. S3). The AUC was calculated for each nasospheroid (see *Methods*) and confirmed no significant differences between C1 and C2 FSK-trajectories (*p* = 0.5205; t = 60 min; t-test) (results of slopes are shown in Supplementary Information). FSK-induced shrinking in nasospheroids was reverted when CFTR inhibitor was added (Supplementary Fig. S4). This indicates that changes observed are depending on CFTR fluid transport.

### Comparison of GraphPad Prism statistical results with a newly developed logarithmic model

Statistical results were obtained by GraphPad Prism software that is widely used for scientist statistical experiments. However, we established a new model with logarithmically transformed measurements without size-adjustments (see *Methods*) to validate results obtained with GraphPad Prism. We then compared results obtained after studying WT nasospheroids by both C1 and C2 with different stimulation (vehicle administration DMSO or FSK). Results were in concordance. Statistical information and values written in *Results* were conducted using GraphPad Prism software. Results obtained with the logarithmic model are included as Supplementary Information. Whilst the logarithmic model allows detailed resolution in significance of the test (*p* value), GraphPad Prism approximates the *p* value when the sample is large (more than 100 values)^33^.

Because we did not report statistical differences between C1 and C2 in FSK responses, we combined results from both WT nasospheroids for the next studies.

### Time and response variability after CFTR activation in WT nasospheroids

A total of 288 nasospheroids from seven non-CF patients were analyzed: 110 with DMSO and 178 in presence of FSK. Differences between vehicle and FSK were observed at minute 10 after the administration of the compounds, and response continued up to 60 min when experiments were completed (FR, and AUC ****p < 0.0001 t-test) (Fig. 1b) (results of slopes are shown in Supplementary Information). These differences increased over time (t = 10 min *p* = 0.008, t = 35 min *p* = 0.0004, t = 70 min *p* = 0.0002) and were more clearly captured using the model with logarithmically transformed measurements than with GraphPad Prism (see *Methods*).

**Figure 2:**
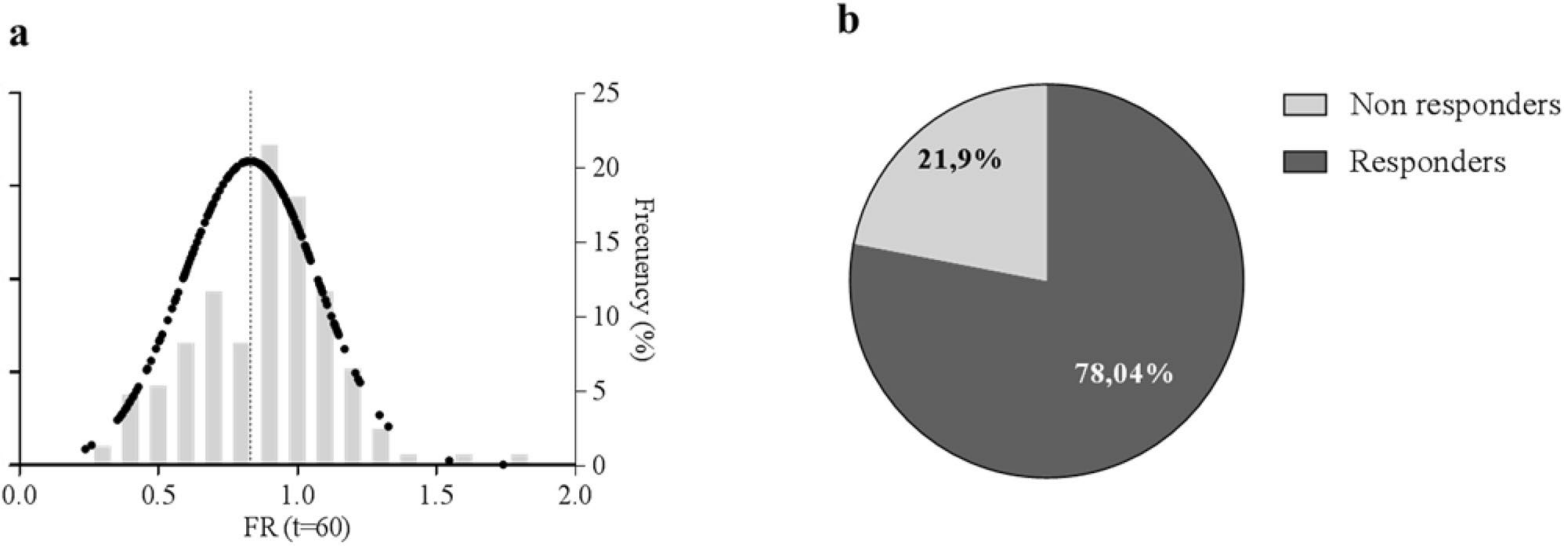
Distribution of FSK responses in WT nasospheroids. (**a**) WT nasospheroids present variability in FR values after 60 min FSK-incubation. Black dots show Gaussian distribution of FR values; right Y axis shows the frequency (%) of FR ranges. Each column represents 0.1 FR interval ranging from 0.2 to 1.7. The dotted line shows WT mean response to FSK (FR = 0.803). (**b**) Distribution of nasospheroids according to their response depending on their FR at t = 60 min. FR ≤ 1 was set for *responders* and FR > 1 for *non-responders*. Graphic created with GraphPad Prism version 6^33^.

Analysis of WT nasospheroids after CFTR activation by FSK differentiated responders and non-responders according to shrinking (Fig. 2a). Classification was based on FR values at t = 60 min: nasospheroids presenting FR > 1 were considered *non-responders* while those with FR ≤ 1 were defined as *responders*. From the 178 FSK-stimulated nasospheroids, we finally studied 173 from the seven WT subjects. We determined that ≈ 80% (135/173) were FSK-responders (Fig. 2b). We categorized responders depending on FR values. Nasospheroids with an FR below the WT mean (FR < 0.803) were considered high responders. Thus, we observed a reduction in area ≥ 20% of their initial size. Following this criterion, 67 of 135 (49.63%) responders were categorized as high responders and 68/135 (50.37%) were classified as low responders (reduction < 20% of its initial area). When studying nasospheroids from C1 and C2 separately, C2 showed a slightly higher proportion of non-responders (~ 30%) in comparison with C1 (~ 20%).

### Potentially all spheres are suitable for analysis independently of their baseline size and condition

**Figure 3:**
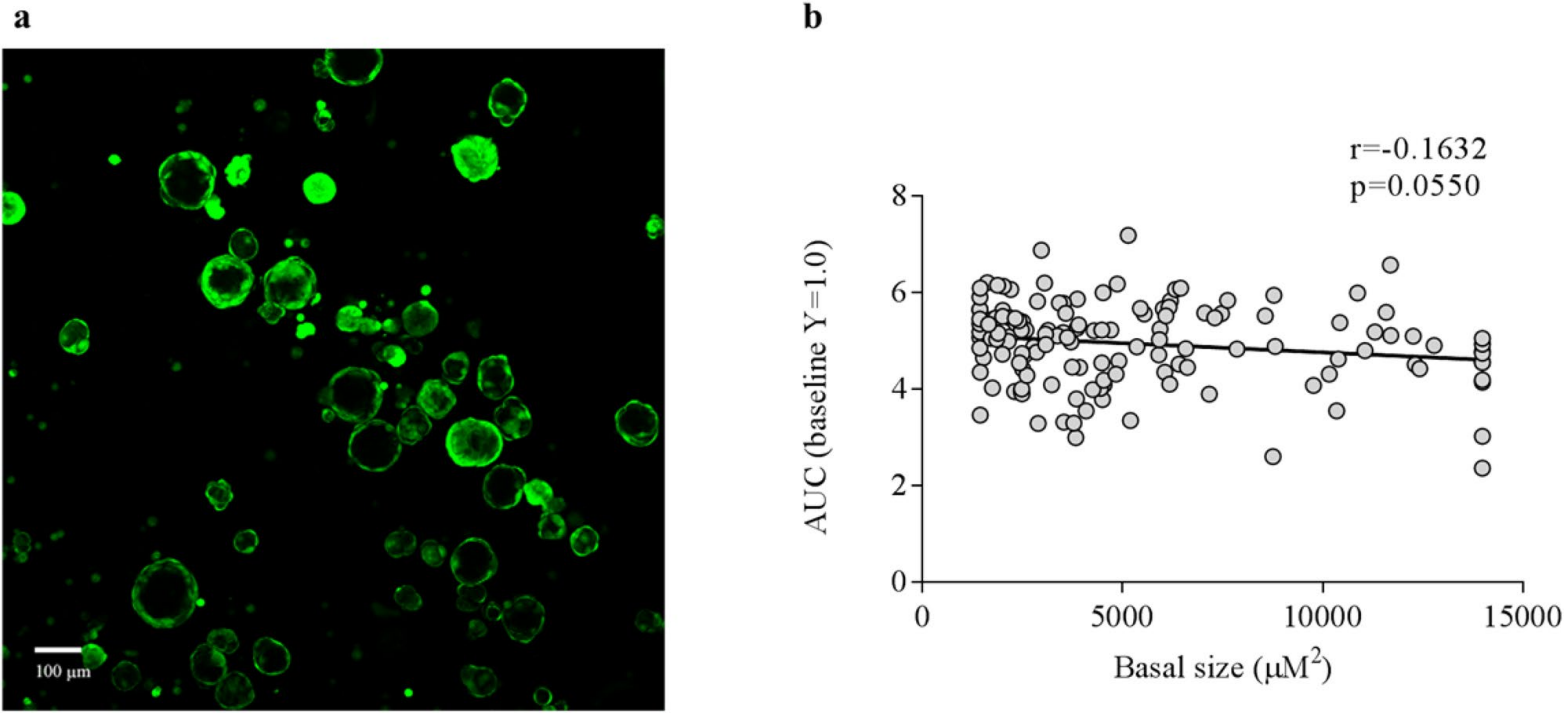
No correlation between basal size of nasospheroids and degree of response after 60 min of FSK incubation. (**a**) Nasospheroids showed diversity basal sizes. (**b**) After excluding non-responders nasospheroids (FR > 1 at t = 60), we matched basal size with AUC values (after CRA analysis). After 60 min of FSK activation in WT nasospheroids, we observed no correlation between the two parameters (basal size and FSK response). Similar results were observed after analysing FR (r =  − 0.1216, *p* = 0.1537) and slopes (results showing slopes of the same experiments are available in Supplementary Information). Results were a combination from spheres grown in conditions 1 and 2. Image taken with confocal microscope LSM780 Zeiss, scale bar created with ImageJ/FIJI ^31^ and graphic created with GraphPad Prism version 6^33^.

The degree of the FSK-response in WT may be influenced by the basal size of nasospheroids. The starting size values of nasospheroids were highly variable (Fig. 3a), ranging from 350 to 53,807 µm^2^ (mean ± SD = 7076.762 ± 6304.907). First, we windsorized the basal size’ values to obtain a more adjusted correlation test. Basal size data was manually windsorized as follows: 5% of the top and 5% of the bottom data was replaced by the maximum or minimum of the values to the nearest extreme. We then performed a Spearman correlation test. In the analysis, we excluded the low proportion of non-responder nasospheroids. After studying 138 nasospheroids, we found no significant correlation when pairing FR values (t = 60 min) and basal sizes (r =  − 0.1216, *p* = 0.1537). We then calculated AUC for each nasospheroid (see *Methods*) (t = 60 min) and showed no statistical correlation between response and size (AUC r =  − 0.1632, *p* = 0.0550) (Fig. 3b), (results of slopes are shown in Supplementary Information).

### Analysis of inner fluid reservoir areas as an effective indicator to study CFTR functionality

**Figure 4:**
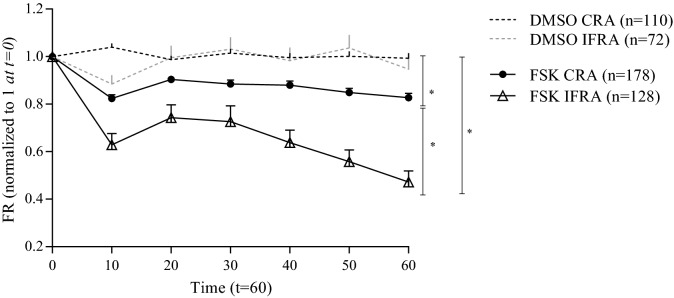
Cross-sectional areas (CRA) and inner fluid reservoir areas (IFRA) analysis of WT nasospheroids from different healthy WT subjects. Spheres were analyzed by live-cell imaging after DMSO or FSK incubation. Dots and triangles show mean FR values up to 60 min. Bars represent SEM. Both CRA and IFRA results present significant differences between the DMSO and the FSK stimulus. T-test also showed statistically different results for CRA and IFRA (*p = 0.0316; paired t-test). Calculated AUC also showed significant differences between CRA and IFRA trajectories (****p < 0.0001). Similar results were observed after analysing slopes (results showing slopes of the same experiments are available in Supplementary Information). Results were a combination from spheres grown in conditions 1 and 2. Graphic created with GraphPad Prism version 6^33^.

When exploring alternative analysis to better evaluate nasospheroid behavior after CFTR stimulation, we observed that *responder* nasospheroids transported fluid from the interior to the media bath, leaving the inner fluid reservoir areas (IFRA) virtually empty in some cases. We then quantitated IFRA of WT nasospheroids after FSK administration up to 60 min. IFRA responders (FR ≤ 1 at t = 60 min), represent almost the 90% of the total samples analyzed (n = 114/128). A comparison between CRA and IFRA responses showed a higher percentage of reduction in the inner parts: CRA (t = 60 min) = 0.805; IFRA (t = 60 min) = 0.473 (Fig. 4) (FR paired t-test *p = 0.0313; AUC ****p < 0.0001 unpaired t-test) (results of slopes are shown in Supplementary Information). Then, we investigated whether IFRA could be more sensitive than CRA to study CFTR functionality and to differentiate CF subjects from the WT group. No basal-size significant differences in IFRA were observed when comparing non-CF and CF nasospheroids (*p* = 0.6459 t-test) (Supplementary Fig. S5a). After FSK treatment, IFRA areas facilitated the recognition of differences between the WT group and CF subjects (*p = 0.0313; paired t-test) (Supplementary Fig. S5b). We also assessed the baseline luminal ratio (BLR) ^32^, IFRA:CRA, and significant differences were also observed between WT group and CF group (*p = 0.0222 t-test).

**Figure 5:**
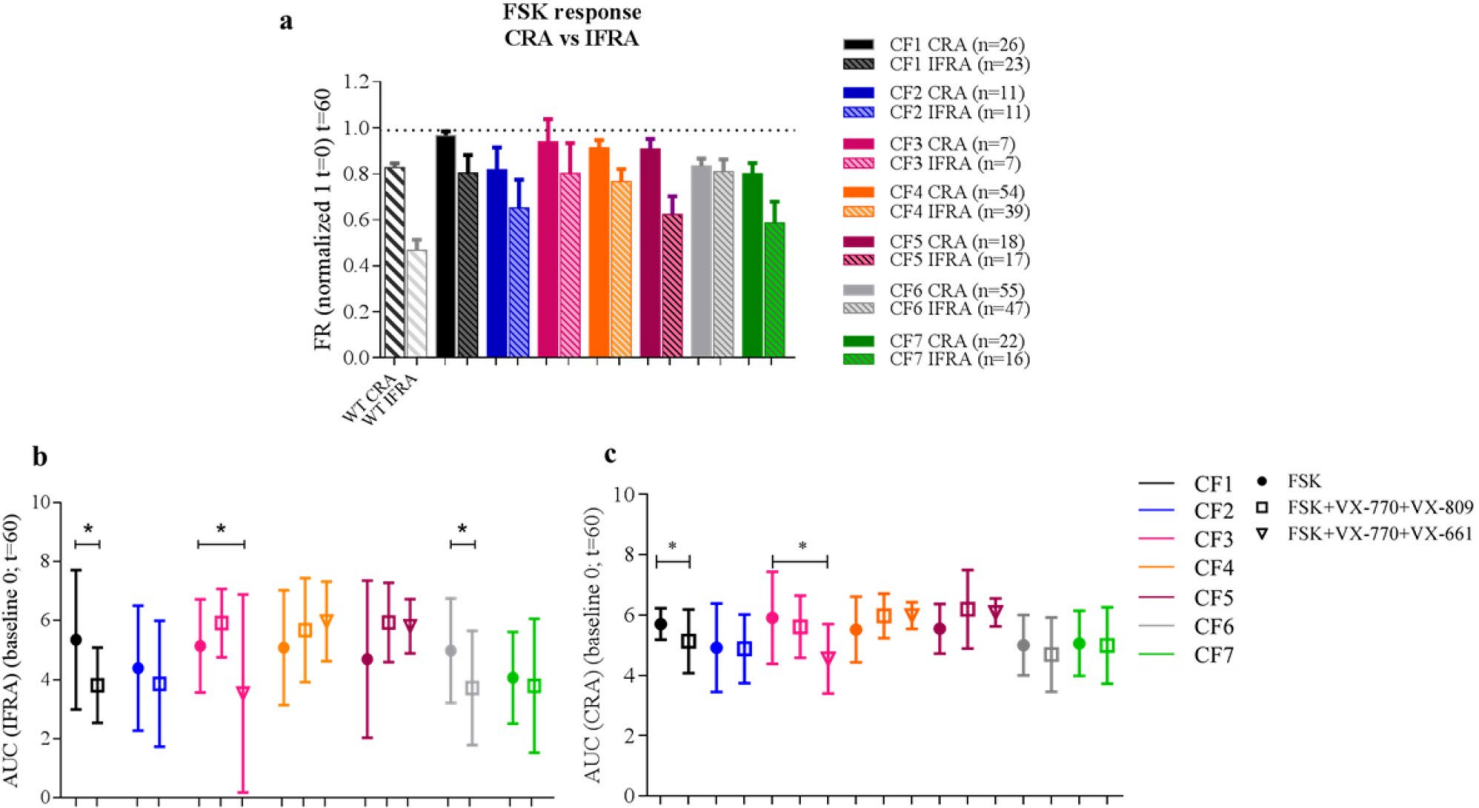
Inner fluid reservoir areas are more sensitive to categorize between healthy and CF responses after FSK-incubation. Both CRA and IFRA areas were studied in WT and CF subjects after 60 min following FSK-incubation. Each CF subject is represented by a different color. All CF nasospheroids were grown under condition 1. (**a**) CRA and IFRA DMSO CF values (t = 60) are represented by a dotted line. CF1 and CF2 unrelated patients share the same genotype (F508del/2183AA>G) but show significant different FSK responses (****p < 0.0001) in IFRA analysis. Columns of CF patients represent mean FR values. Bars represent SEM. AUC values after IFRA (**b**) and CRA (**c**) assessment were determined by GraphPad Prism (baseline Y = 0) at t = 60 min. Dots represent mean AUC values from FSK-incubated nasospheroids. Empty squares represent mean AUC values of VX-770 + VX-809-treated nasospheroids. Inverted triangles show mean AUC values from VX-770 + VX-661-treated nasospheroids. Bars represent standard deviation (SD). Significant differences (*p* < 0.05) within VX-treated and FSK-treated nasospheroids are shown. VX-incubation was always combined with FSK, Amiloride and IBMX. Graphic created with GraphPad Prism version 6^33^.

Differences in FSK response were also observed between CF patients (n = 7) with different genotypes (Table 1). Two patients (CF1 and CF2) sharing the same CF genotype (F508del/2183AA>G) showed differences in CFTR functionality after FSK stimulation (Fig. 5a). The CF1 patient had severe lung disease in clinical assessment (Table 1) and showed almost no shrinkage in comparison with the sample from the CF2 patient, which had milder lung disease (Fig. 5a, Supplementary Fig. S6).

### Differential responses of nasospheroids from CF patients after combinatorial treatment of CFTR modulators

**Figure 6:**
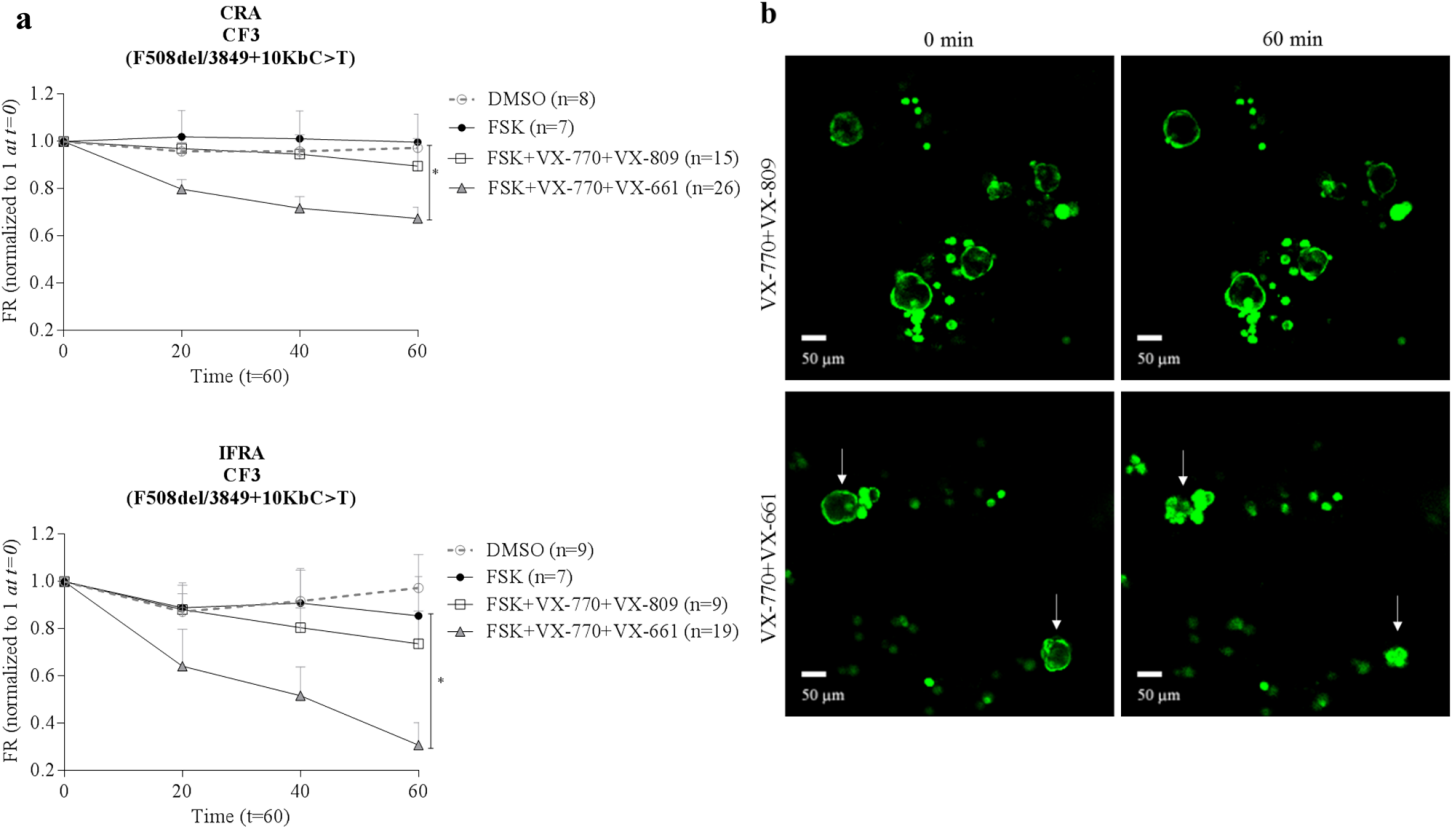
Pharmacological response in nasospheroids from patient CF3. (**a,b**) Patient CF3, presenting F508del/3849 + 10KbC>T genotype, showed no significant differences between FSK and VX-770 + VX-809 administration (CRA *p* = 0.2978, IFRA *p* = 0.5848) after 60 min of follow-up while a significant response was seen in presence of the VX-770 + VX-661 combination in comparison with only FSK (CRA * *p* = 0.0104, IFRA **p = 0.0047) (**a,b**). Bars represent SEM. VX-incubation was always combined with FSK, Amiloride and IBMX. All CF nasospheroids were grown under condition 1. Image taken with confocal microscope LSM780 Zeiss, scale bar created with ImageJ/FIJI ^31^ and graphic created with GraphPad Prism version 6^33^.

To assess the pharmacological restoration of CFTR function in our seven CF subjects, we tested several combinatorial regimens of FSK + CFTR modulators: VX-770 + VX-809 and VX-770 + VX-661. All CF nasospheroids were grown in C1 conditions according to previous results seen in WT samples. After performing three types of data analyses (FR, AUC and slopes) to examine CRA and IFRA we found that some patient samples showed a drug-induced response while others presented similar values in both FSK and VX-treated conditions (Fig. 5b, c, Fig. 6, Table 1, Supplementary Fig. S6). In patient CF6 (F508del/F508del) nasospheroids decreased in size after treatment but a statistically significant VX-770 + VX-809 response was only seen after IFRA assessment (FR values *p = 0.0170, AUC *p = 0.0105 in comparison to FSK-treated nasospheroids) (Fig. 5b, Supplementary Fig. S6).

## Discussion

Here, we validated and explored the reproducibility of a functional CFTR assay in nasospheroids ^30^, studying seven WT subjects and seven CF patients presenting a variety of genotypes and phenotypes. We performed a series of modifications and changes aiming to improve the accuracy of the protocol and the following analysis. First, we made technical adjustments to increase the number of observations (*n*) for statistical power. Second, we performed a wider analysis of nasospheroids (CRA and IFRA, outer and inner part respectively) which helped to categorize the results and observations. And third, we implemented the use of a simple statistical analysis by GraphPad Prism to examine nasospheroids responses. We found that the results of our mixed linear statistic model (without size-adjustment) in comparison with those obtained by GraphPad Prism were the same for both models. In view of this equivalence, the functional assay using nasospheroids could be a more accessible and reproducible approach in research laboratories.

Technical adjustments were explored using WT nasospheroids. By embedding nasospheroids in Matrigel, we avoided the inconvenience of suspension cells running outside the focus during live-cell imaging experiments (t = 60 min). This allowed the in vitro study of hundreds of nasospheroids formed in a multi-well plate. By immobilizing the nasospheroids in culture, we increased number of cells by up to 20-fold during microscopy studies ^30^. The overall responses were therefore evaluated more categorically. WT nasospheroids were then characterized as responder or non-responder (classified depending on FR results). We determined that the overall frequency of responders was around 80% for each experiment when studying CRA and almost 90% when analysing IFRA. In our hands, we observed that the proportion of non-responders tends to be higher in C2 nasospheroids (~ 30%) in comparison to C1 nasospheroids (~ 20%). Although we were not able to observe cilia facing inward in the studied nasospheroids, we cannot discard that some cells could present apical-inside membranes after a prolonged Matrigel-time exposure. However, mean FSK response was not different between both groups. For simplicity, we have grown all CF nasospheroids in C1.

In our analysis, levels of responses were independent of the baseline size of nasospheroids. The previous study ^30^ hypothesized that nasospheroids with similar baseline sizes would show a similar degree of response and should be investigated. According to our results, all spheres were potentially acceptable for analysis. Heterogeneous responses observed in a proportion of our cells might be explained by different CFTR expression levels in nasospheroids of the same individual.

While exploring alternative analysis to CRA, we performed measurements of inner areas, which we found to be more sensitive than the CRA to discriminate between WT and CF FSK-responses. In our model, nasospheres grow with the luminal surface facing outward and shrink following CFTR activation. Brewington and colleagues reported the analysis of inner areas in another nasal spheroid model that swell after FSK-stimulation ^35^. Dekkers et al. established the analysis of inner parts for analogous CFTR functional studies in intestinal organoids to discern between WT and CFTR phenotypes ^19^. They reported that organoids from severe CF patients tended to have a more cystic shape and smaller inner areas than those from patients with milder illness and WT subjects. A recent report ^32^ also showed differences in basal lumen size in nasal spheres (with inward apical orientation) between CF and non-CF samples and reported that the BLR value (IFRA:CRA) can distinguish between the WT group and CF group. In our study, we did not find any significant differences between basal inner areas of WT group and CF group but our BLR values indeed showed significant differences. This suggests that BLR may be a useful measure to differentiate CF from WT nasospheroids regardless of the culture condition.

When studying CF patients, we found that not all patient samples showed a drug-induced response, an observation that has been previously reported ^30^. Two CF subjects (CF1 and CF2) who shared the same genotype (F508del/2183AA>G), showed different responses after FSK stimulation. Interestingly, patient CF1, who showed almost no shrinking, had more severe pulmonary disease than patient CF2 (Table 1). In fact, patient CF1 showed the lowest levels of CFTR activation after FSK (Fig. 5a). Differences in CFTR functionality among patients with the same genotype have also been observed after FIS assay using intestinal organoids ^21^. These differences should be considered in the context of genomic background or the effect of modifier genes ^36^.

Functional CFTR analysis by nasospheroids was also effective to determine the effect of in vitro treatment using a combination of CFTR modulators. We treated nasospheroids from seven CF patients with the combination VX-770 + VX-809, approved by the FDA ^37^ and the EMA ^38^, for patients with specific *CFTR* genotypes. In addition, we studied several patients who were possibly in vitro responders to the VX-770 + VX-661 combination also approved by FDA and EMA ^9,39,40^. An interesting example was patient CF3 (F508del/3849 + 10KbC>T), who showed a significant response to VX-770 + VX-661 (FR, *p = 0.0104 CRA analysis; **p = 0.0047 IFRA analysis) but not to VX-770 + VX-809 (FR, *p* = 0.2978 CRA analysis; *p* = 0.5848 IFRA analysis) (see Fig. 6). This result is consistent with the current approved genotype indication of VX-770 + VX-661 (SYMDEKO/SYMKEVI) for patients carrying variants such as our CF3 patient ^9^. Correlations between in vitro results in treated nasospheroids and in vivo clinical improvements might be necessary to predict individual responses.

For a systematic analysis of the response to CFTR modulators, we used three variables (FR, AUC and slopes) and two different areas of study (CRA and IFRA). Results between the three variables were equivalent; showing similarities when determining which patients present a significant drug-induced volume reduction. In comparison with slopes, AUC analysis offers the advantage of not assuming linear responses ^30^. AUC values are commonly used in analogous CF in vitro assays using intestinal organoids ^17^ and may be of interest to compare results of a given sample that can be obtained with both methods (organoids and nasospheroids).

In some CF samples we found differences between the results of CRA and IFRA. For example, CF6 with an F508del/F508del, a genotype that usually responds to VX-770 + VX-809 (ORKAMBI) ^41–43^, only showed an in vitro significant response when analysing FR and AUC in IFRA trajectories. Thus, the study of inner areas, which show a wider range of shrinking than CRA, may result in a more robust categorization of doubtful cases.

Future studies using nasospheroids may include an extended time for live-cell observations in some cases with rare CF genotypes ^44,45^ and testing of recently approved modulator drugs, such TRIKAFTA/KAFTRIO ^46^.

The present modifications of the protocol help to improve the applications of nasospheroids to perform functional CFTR studies. This technique, however, is not without disadvantages. Differentiated nasal cells do not duplicate once nasospheroids are fully formed. Thus, the number of structures obtained is limited. Additionally, nasospheroids do not recapitulate in vivo structures, as they are an artificial product formed in culture. Very recently, a more complex protocol using epithelial nasal cells was described as a potential tool to study CFTR ^32^ reinforcing the inherent  value of nasal epithelium in the study of CF disease. Moreover, it has been showed that in vitro nasal cells can recapitulate functional CFTR characteristics of in vitro bronchial cells for individualized CFTR analysis ^25^. Nasal models present the advantage of enable the study of CFTR airway functionality in an easily accessible sample, unlike bronchial epithelial models.

In conclusion, we present a series of modifications and improvements to measure CFTR functionality in nasospheroids. This simple and affordable, non-invasive approach provides CFTR read-out within a week. Our data indicates that this  improved method can be used as an initial or complementary tool to define CFTR airway epithelial function and to predict which patients will respond to modulator therapy.

## Supplementary Information

Supplementary Information 1.

Supplementary Video 1.

Supplementary Video 2.

## Data Availability

All data generated or analyzed during this study are included in this published article (and its Supplementary Information files).
