# Supplementary material for: Validation of nasospheroids to assay CFTR functionality and modulator responses in cystic fibrosis
Source: Sci Rep. 2021 Jul 30;11:15511. doi: 10.1038/s41598-021-94798-x (PMC8324871; doi:10.1038/s41598-021-94798-x)
Supplement: Supplementary file 1 — Supplementary Information 1. [file 41598_2021_94798_MOESM1_ESM.docx]

**Online Supplementary Information**

**Validation of nasospheroids to assay CFTR functionality and modulator responses in cystic fibrosis**

Maite Calucho, Silvia Gartner, Paula Barranco, Paula Fernández-Álvarez, Raquel García Pérez, Eduardo F. Tizzano

**Supplementary Results**

*Logarithmic Model*

**Supplementary Table S2** shows the variables included in the logarithmic model. Applying the initial logarithmic model (see **Supplementary Table S3**), statistical differences were found between stimulus FSK and DMSO (p=0.0047), and stimulus through time “Stimulus:T” (p=0.0001). No statistical differences were found when comparing measures of conditions C1 and C2 (p=0.4684) and neither “Condition:T” (p=0.8892). Then, the variable “Condition” was eliminated from the initial model (see **Supplementary Table S2 and S3**). When removed “Condition” from the model, differences between “Stimulus” and “Stimulus:T” were maintained statistically different (p=0.0053; p=0.0001).

When studying the logarithmically transformed relative growth (“Y2”) (see **Supplementary Table S3**), statistical differences were found between “Stimulus” and “Stimulus:T” (p=0.0009, 0.0216). No statistical differences were found between “Condition” and “Condition:T” (p=0.4456, p=0.5196). Then, variable “Condition” was eliminated from the model (see **Supplementary Table S3**). Again, statistical differences were observed between “Stimulus” and “Stimulus:T” (p=0.0008, p=0.0204) and those increase over time (p=0.0087, t=10, p=0.0004 t=35 and p=0.0002 t=70).

*Section “Formation and embedding of nasospheroids for in vivo imaging analysis”*

Slopes were calculated for each nasospheroid and confirm no significant differences between C1 and C2 FSK-trajectories (p=0.1297 t-test; t=60 minutes).

*Section* “*Time and response variability after CFTR activation in WT nasospheroids”*

We observed statistically differences in slopes between DMSO and forskolin administration in WT nasospheroids (slopes p<0.0001 t-test; t=60 minutes).

*Section “Potentially all spheres are suitable for analysis independently of their baseline size”*

We calculated slopes for each nasospheroid (t=60 minutes) and showed no statistical correlation between response and basal size (r=0.04124, p=0.6298).

*Section* “*Analysis of inner fluid reservoir areas as an effective indicator to study CFTR functionality”*

IFRA slopes after FSK in WT nasospheroids showed a higher reduction in comparison with CRA analysis (p<0.0001 t-test; t=60 minutes).

*Supplementary Table S1.* *Classification of CFTR* *pathogenic variants in classes I to VII, their consequences in the CFTR protein and potential therapies*.

| ***CFTR* mutation class** | **Defect in CFTR protein** | **Therapeutic strategy** |
| --- | --- | --- |
| **Class I** | No protein synthesis. mRNA is often degraded | Read-through compounds |
| **Class II** | Protein is retained in the endoplasmic reticulum and is not transported to the apical membrane | Modulators (correctors and potentiators) |
| **Class III** | Impaired channel gating. Reduced channel opening probability | Modulators (potentiators) |
| **Class IV** | Changes in protein structure producing low chloride conduction | Modulators (potentiators) |
| **Class V** | Reduced levels of normal protein, often due to alternative splicing. Splicing produces both normal and aberrant CFTR | Modulators (correctors) |
| **Class VI** | Protein instability in the apical membrane leading to rapid CFTR removal | Stabilizers |
| **Class VII** | Gene deletion. No CFTR production | Gene therapy |

*Supplementary Table S2.* This table shows the two main variables (explanatory and response) and subvariables within the two groups that are included in the mixed-linear logarithmic model used to study the area of WT nasospheroids. An explanatory variable is a type of independent variable. A response variable is the focus of a question in the experiment.

| **Explanatory variables** | **ID** | Individual |
| --- | --- | --- |
|  | **ID BLOC** | Consistent replication of a set of nasospheroids |
|  | **ID nasospheroids** | Individual identification of nasospheroids |
|  | **T** | Time points (t0, t10, t20, t30, t40, t50, t60) |
|  | **Stimulus** | Stimulus applied to each sample (DMSO or FSK) |
|  | **COND** | Cell culture condition (*Condition 1* or *Condition 2*) |
| **Response**  **variables** | **Measure** | The value of the measures (µm^2^) |
|  | **Y** | Values of the measure logarithmically transformed: log(measure) |
|  | **Relative growth (MR)** | Ratio of measure between basal measure: measure/initial measure |
|  | **Y2** | Logarithmic of MR: log(measure/initial measure) |

*Supplementary Table S3.* This table presents the logarithmic model with formulas and variations used to study the area of WT nasospheroids*.* The logarithmic model was specifically designed for our study to analyze the area of WT nasospheroids through time. Several model structures were explored to analyze the variation of areas through time. Statistical analysis was carried out using SAS v9.4 and R software v3.1.2.

| **Initial model** | m0 <- lmer (Y ^~^ STIMULUS + COND + T + STIMULUS:T + COND:T + (T\|ID:BLOC), data) |
| --- | --- |
| **Initial model without variable “COND”** | m0 <- lmer (Y ^~^ STIMULUS + T + STIMULUS:T + (T\|ID:BLOC), data) |
| **Y2 model** | m01 <- lmer (Y2 ^~^ STIMULUS + COND + T + STIMULUS:T + COND:T + (T-1\|ID) + (T\|ID:BLOC), data[data$T>0,] ) |
| **Y2 model without variable “COND”** | m11 <- lmer (Y2 ^~^ STIMULUS + T + STIMULUS:T + (T-1\|ID) + (T\|ID:BLOC), data[data$T>0,] ) |

*Supplementary Figure S1. Confocal microscopy images of CFTR detection in suspended WT nasospheroids.* After 7-8 days of cell culture, WT nasospheroids were fixed with paraformaldehyde (PFA) (4ºC, 45 min) [^2^](#_ENREF_2). (**a**) Primary antibody Anti-CFTR (MAB1660-SP; R&D Systems) was incubated O/N, 4ºC at 1:10 concentration [^3^](#_ENREF_3). Secondary antibody used was Alexa Fluor 488 goat anti-mouse IgG (115-545-003; Jackson Immuno Research) diluted 1:500 and incubated 3 hours at 4ºC. DAPI (D1306; ThermoFisher) was used at 1:1000 to stain the nucleus. Cells were mounted in 8-wells chambers (iBidi) with fructose-glycerol clearing solution [^2^](#_ENREF_2). (**b**) The same protocol was followed for the negative control but without incubation of primary antibody anti-CFTR. Images show one z-stack and were taken by a confocal microscope. Scale bars: 20 µm.

*Supplementary Figure S2. WT nasospheroids embedded in Matrigel under C1 and C2 present cilia facing outside.* Right panel shows non-fixed C1 (**a**) and C2 (**b**) WT nasospheroids stained with Cell Mask Orange 1X (red; cilia and plasma membrane) and Calcein Green AM 8 µM (green; live cells) [^1^](#_ENREF_1) and left panel shows brightfield. Images show one z-stack and were taken by a confocal microscope. Scale bars: 20 µm.


*Supplementary Figure S3. WT nasospheroids in suspension present similar values than WT nasospheroids embedded in Matrigel after FSK treatment.* WT nasospheroids from two WT individuals grown in suspension and never embedded in Matrigel. After 8 days in culture, cells were collected, centrifuged (400 g, 5 min) and seeded in black 24 well plates. Cells were incubated with FSK, IBMX and Amiloride as described in the article. No significant differences were reported after 60 minutes of FSK treatment between suspension nasospheroids and Matrigel-embbeded naosospheroids (C1 and C2) (p=0.9839, p=0.5138; t-test).

*Supplementary Figure S4. WT nasospheroids revert shrinking when are treated with FSK plus CFTR inhibitor.* Nasospheroids from one healthy WT were grown in suspension and embedded in Matrigel the day before the live-cell imaging (C1). WT nasospheroids are shown to respond to FSK after 60 minutes of live-cell imaging observations and shrinking is reverted when 10 µM CFTR inhibitor-172 was incubated (3 hours) prior to FSK, Amiloride and IBMX (FSK vs CFTR inhibitor *p=0.0313 paired t-test).[^1^](#_ENREF_1) Bars represent SEM.

*
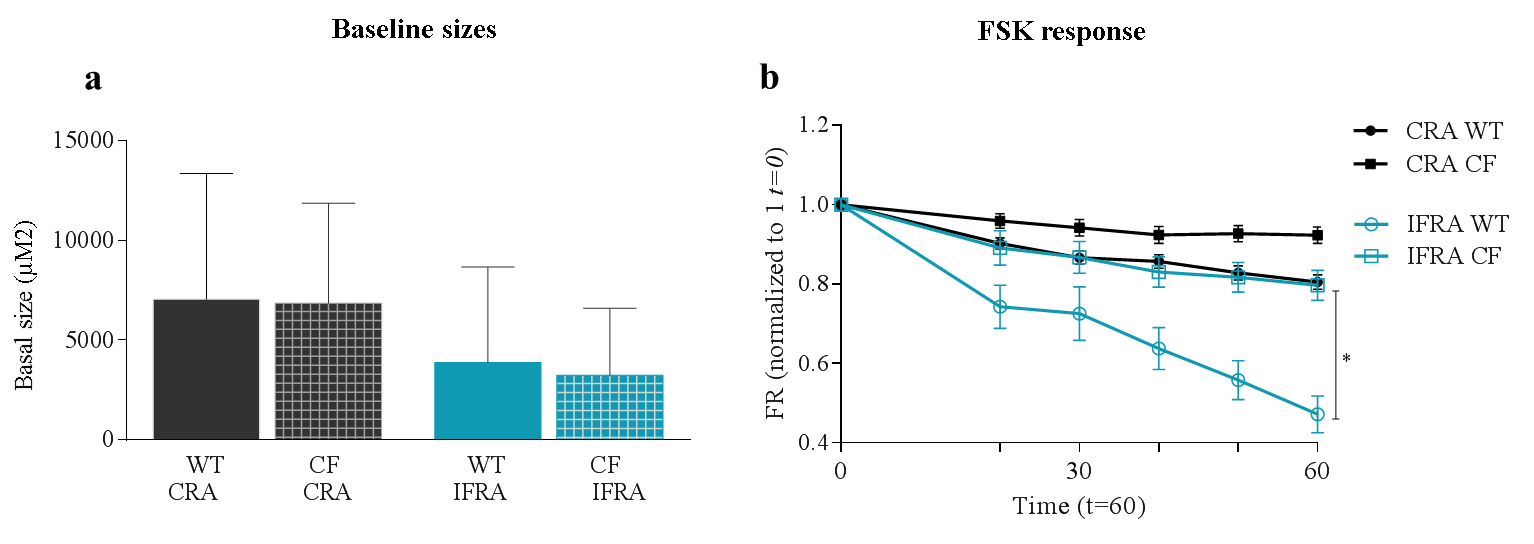
Supplementary Figure S5.* *CRA and IFRA sizes in WT group and CF group.* **(a)** No basal-size differences were seen between nasospheroids from WT group and CF group (CRA p=0.2443, IFRA p=0.6459; t-test). Columns represent mean basal sizes and bars represent SD. **(b)** After FSK treatment, IFRA analysis is more sensitive to differ between WT and CF group (CRA p=0.0625, IFRA *p=0.0313; paired t-test). Bars represent SEM. All CF nasospheroids were grown under condition 1.

*Supplementary Figure S6.* *IFRA analysis in nasospheroids from seven CF subjects treated with FSK alone and FSK+modulators (VX-770+VX-809 and VX-770+VX-661).* FR mean values every 10 minutes and over 60 minutes are represented by dots, squares or triangles. VX-incubation was always combined with FSK, Amiloride and IBMX. Bars represent SEM. All CF nasospheroids were grown under condition 1.

*Supplementary Video S1. WT nasospheroid embedded in Matrigel under C1 showing cilia facing outside.* Images were taken consecutively by a confocal microscope every one second up to one minute. The video is created with ImageJ/FIJI by combining all the images at 7fps frame rate. Video shows one z-stack. Scale bar: 20 µm.

*Supplementary Video S2. WT nasospheroid embedded in Matrigel under C2 showing cilia facing outside.* Images were taken consecutively by a confocal microscope every one second up to one minute. The video is created with ImageJ/FIJI by combining all the images at 7fps frame rate. Video shows one z-stack. Scale bar: 20 µm.

**References Online Supplementary Information**

1 Guimbellot, J. S. *et al.* Nasospheroids permit measurements of CFTR-dependent fluid transport. *JCI Insight* **2**, doi:10.1172/jci.insight.95734 (2017).

2 Dekkers, J. F. *et al.* High-resolution 3D imaging of fixed and cleared organoids. **14**, 1756-1771, doi:10.1038/s41596-019-0160-8 (2019).

3 Carvalho-Oliveira, I. *et al.* CFTR localization in native airway cells and cell lines expressing wild-type or F508del-CFTR by a panel of different antibodies. *J Histochem Cytochem* **52**, 193-203, doi:10.1177/002215540405200207 (2004).
